# Gastric protective effect of *Alpinia officinarum* flavonoids: mediating TLR4/NF-κB and TRPV1 signalling pathways and gastric mucosal healing

**DOI:** 10.1080/13880209.2022.2152058

**Published:** 2022-12-21

**Authors:** Kaiwen Lin, Tang Deng, Huijuan Qu, Hongya Ou, Qifeng Huang, Bingmiao Gao, Xiaoliang Li, Na Wei

**Affiliations:** a School of Pharmacy, Hainan Medical University, Haikou, China; b Hainan Women and Children’s Medical Center, Haikou, China; c First Affiliated Hospital of Hainan Medical University, Haikou, China

**Keywords:** Gastric ulcer, gastric healing, anti-inflammatory, gastric epithelial cell, RNA-sequencing

## Abstract

**Context:**

Our previous studies have found that total flavonoid of *Alpinia officinarum* Hance (Zingiberaceae) (F.AOH) had protective effects on gastric ulcer (GU).

**Objective:**

To investigate the protective mechanism of F.AOH on acetic acid-induced chronic GUs in rats and ethanol-induced GES-1 cells damage.

**Materials and methods:**

*In vivo*: Gastric damage was induced in SD rats by administering acetic acid after oral treatment with F-AOH at 54, 27 and 13.5 mg/kg (2 weeks of continuous gavage). After a comprehensive evaluation of rats’ serum and gastric tissue-related indicators, gene transcriptome sequencing, qPCR and Western blotting were used to investigate the mechanism further. *In vivo*: GES-1 cells were incubated with F-AOH (8, 4 and 2 μg/mL) for 16 h and treated with 7% ethanol for 4 h. Transwell and flow cytometry were employed to detect migration and apoptosis of cells.

**Results:**

F.AOH effectively reduced the area of GUs in rats (from 11.2 ± 1.89 to 2.19 ± 0.95), reversing ethanol-induced cells apoptosis (from 23 ± 1.3 to 8.11 ± 0.93%). It also inhibited the expression of endothelin-1 (ET-1) and iNOS proteins, decreasing the levels of TNF-α IL-6 in serum, improving oxidative stress levels and increasing the expression of Bcl-2/Bax dimer genes. In addition, 4005 differentially expressed genes between the acetic acid model and the drug groups. Through experimental verification, F.AOH can inhibit the activation of TLR4/NF-κB signalling pathway and TRPV1 receptor.

**Conclusions:**

F.AOH, as an effective gastric protective plant component, had potential therapeutic value in anti-inflammatory pain and antioxidative stress gastrointestinal diseases.

## Introduction

Gastric ulcers (GUs) occur in the stomach lining or deeper in the stomach, mostly due to the imbalance between the body’s defence system and external pathogenic factors (Asali et al. 2018). The pathogenesis of GU and the disease mechanism in the body are multi-factorial and complex. Therefore, further exploration of the mechanical signal transmission in the pathogenesis of GU can better explain the complex pathological process of GU and take more effective treatment measures.

There are many kinds of drug treatments for GU in clinical medicine. However, their use is unable to treat GU completely, and it is easy to increase the risk of recurrence, which is often accompanied by adverse reactions, including effects on osteoporotic fracture, rare nephrotoxicity and hepatotoxicity (Yu et al. 2017). Accordingly, investigating a novel and effective drug for treating GU has great commercial prospects and medical strategies. In recent years, with the development and utilization of medicinal plants, a large amount of evidence indicates that medicinal plants are more effective in treating digestive tract diseases and reducing the adverse drug reactions and the recurrence rate of GU (Ardalani et al. 2020). In Asian and African countries, the dried rhizomes of *Alpinia officinarum* Hance (Zingiberaceae) are often made into a decoction or added to other foods as pharmaceutical supplements homologous to medicine and food to treat various diseases such as cough, malaria, diabetes and digestive system diseases (Abubakar et al. 2018).

F.AOH is one of the most biologically active compounds in *A. officinarum*. Our previous research results demonstrated that F.AOH could inhibit NF-κB/CO_2_ pathway, improve gastrointestinal motility and prevent alcohol-induced GU. However, the specific target mechanism has been determined yet (Lin et al. 2020).

To further clarify the anti-GU effect and mechanism of F.AOH, this study established a rat model of acetic acid-induced GU and an ethanol-induced acute injury model of human gastric mucosal epithelial cells to simulate the environment of chronic GU disease and gastric mucosal epithelial cell injury, respectively. The evaluation and mechanism research of F.AOH anti-GU through relevant GU characteristic indexes include measurement of ulcer area, histopathological evaluation, expression and release of inflammatory proteins and inflammatory factors, and antioxidative stress effect. Transwell experiments and flow cytometry were used to detect cell repair and apoptosis. Simultaneously, we used mRNA-seq, western blotting and qPCR to screen and verify the expression of related genes and proteins and further explore the gastric protective mechanism of F.AOH.

## Materials and methods

### Plant materials, extraction preparation and structure analysis

In October 2017, the rhizome of *A. officinarum* was collected (Haikou city, Hainan Province, China) and identified by Professor Zeng Niankai (Hainan Medical University). Some samples were stored in the Laboratory of Natural Phytomedicinal Chemistry of Hainan Medical University (specimen number: 20171024). Subsequently, the composition and structure of flavonoid compounds were extracted and analysed using laboratory-validated extraction methods (a kilo of A. *officinarum* was refluxed with 80% ethanol for 1 h and concentrated to 40% under reduced pressure. Then, the extract was purified with AB-8 macroporous. The ethanol elution fraction was subjected to silica gel column chromatography and eluted with a petroleum ether-ethyl acetate gradient. The fractions were subjected to gel column chromatography and eluted with methanol to obtain F.AOH), ultra-high performance liquid chromatography diode array detector, and mass spectrometer analysis (the ultra-high performance liquid chromatography-diode array detector-mass spectrometry (UHPLC-DAD-MS) data were obtained from an Agilent 1290 Infinity series UHPLC system with a diode array detector and an Agilent 6120 quadrupole mass spectrometer. The mass spectrometer contained a dual atmospheric pressure chemical ionization (APCI) and electrospray ionization (ESI) interface.) (Lin et al. 2020), and flavonoids were then stored in the refrigerator at −20 °C for subsequent analysis.

### Animals

A total of 60 Sprague-Dawley female rats, 6–7 weeks in age (180–200 g) were obtained from Changsha Tianqin Biotechnology Co., Ltd. (Changsha, China). The rats were free to eat standard rodent feed and drink pure water during a controlled cycle (12 h light/dark cycle, room temperature 22–24 °C, relative humidity 40–60%). All animal experiments were carried out according to the requirements of the Animal Experimental Ethics Committee of Hainan Medical University (no. hy2020061701, Haikou, China) and the guidelines for the Care and Use of Experimental Animals.

### Cell culture

Human gastric epithelial cells (GES-1) were cultured at 37 °C in a cell incubator with 5% CO_2_. Cell culture medium: RPMI 1640 culture medium (Gibco, Brooklyn, NY) supplied with 10% heat-inactivated foetal bovine serum (FBS) (Biological Industries, Beit Haemek, Israel), 100 U/mL of penicillin and 100 L/mL of streptomycin (Beyotime, Nanjing, China). Our previous study established the optimal concentration and time of ethanol-induced acute gastric mucosal cell injury and the drug intervention concentration (Lin et al. 2020). On this basis, a transwell assay was performed to evaluate the cell migration ability. Flow cytometry was used to detect the cell apoptosis rate, and Western blot and qPCR techniques were used to detect the expression of Bcl-2/Bax/caspase-3 in the apoptosis pathway.

### Animal experimental design

After two days of environmentally adaptive feeding, the rats were randomly divided into the normal group (*n* = 10, contacting the stomach wall with saline) and the surgical model group (*n* = 50, burning the stomach wall with 80% acetic acid). A 24 h before the establishment of the model, all rats fasted and drank freely. The GU model is slightly modified based on the reference method (Okabe et al. 1971). Under continuous isoflurane inhalation anaesthesia, a 2 cm incision was made at the lower left of the xiphoid process of the abdomen, and the stomach tissue was carefully exposed to the outside of the body. Then, 200 μL of normal saline or 80% acetic acid was injected into a cylindrical hole with a diameter of 0.8 cm, which is close to the gastric antrum of the rat. After 1 min, acetic acid or normal saline was sucked out with a medical cotton swab, and the burn was rinsed slowly with normal saline to prevent acetic acid from stimulating the gastric antrum. Moreover, the muscular layer and skin were sutured successively. Two days after the establishment of GU model, 50 operation model rats were randomly divided into 10 rats in each group. Then, the rats in the operation model group and the normal group (*n* = 10) were divided into the following groups: (1) normal group, (2) acetic acid model group, (3) acetic acid model + ranitidine (RAN) group (100 mg/kg), (4) acetic acid model + low- (13.5 mg/kg), (5) acetic acid model + medium- (27 mg/kg) and (6) acetic acid model + high-dose (54 mg/kg). All rats were treated with corresponding drugs (different concentrations of F.AOH and RAN) or 0.5% carboxymethylcellulose sodium for 14 consecutive days. After 24 h of the last treatment, serum samples were collected from the abdominal aorta under deep isoflurane anaesthesia. Gastric tissue was quickly removed, incised along the greater curvature of the stomach, rinsed with pre-cooled normal saline, fixed on a blue background, and photographed. The ulcer index (UI) was scored. GU was divided into two parts. One part was placed in a 4% paraformaldehyde solution for histopathological analysis, and the other part was stored in a refrigerator at 80 °C for biochemical analysis.

### Determination of the injury index of gastric ulcer

The UI was calculated according to the following equation:

(1) Ulcer index (UI)=a×b 

where *a* is the maximum length of ulcer surface diameter *A* and *b* is the maximum length of the diameter *B* perpendicular to *A* on the ulcer surface.

The ulcer inhibition rates of F.AOH and RAN were calculated according to the following equation:

(2) $$\text{Inhibition}=[({\text{UI}}^{1}-{\text{UI}}^{2})\times 100\%]/{\text{UI}}^{1}$$

where UI^1^ is the ulcer index of the acetic acid group and UI^2^ is the ulcer index of F.AOH or RAN group.

### Histopathological analysis

The gastric tissue was fixed in 4% paraformaldehyde solution, dehydrated with ethanol solution and xylene, embedded in paraffin, cut into sections with a thickness of 3 μm, dewaxed, and stained with haematoxylin–eosin for histopathological examination.

### Enzyme-linked immunosorbent assay

The level of TNF-α, IL-6, glutathione (GSH) and malondialdehyde (MDA) was determined using an ELISA kit (Elisa Biotech, Shanghai, China). Simultaneously, the activities of MPO and SOD were measured according to the test kit instruction provided (Nanjing Jiancheng Bioengineering Institute, Nanjing, China). All tests were performed according to manufacturer’s protocols. The absorbance value was measured on a Microplate spectrophotometer (BioTek Epoch; BioTek Instruments, Inc., Winooski, VT).

### *In vitro* experiment of transwell

In the Transwell experiment, 1 × 10^5^ GES-1 cells were inoculated into the upper compartment of the transwell. After incubation for 24 h, 7% ethanol was added for 4 h to induce GES-1 cells damage. The upper chamber was then transferred to a new 24-well plate. First, the lower chamber was added to a serum-free medium and incubated for 8 h. Then, the upper chamber was transferred to a medium containing 15% FBS for further incubation for 16 h. During this period, a 200 µL serum-free medium was added to the upper chamber of the normal and ethanol groups, and 200 μL F.AOH (2, 4 and 8 μg/mL) and RAN (100 μg/mL) were added to the drug groups, respectively. After a total of 24 h culture, residual cells that failed to pass through the pore membrane in the upper chamber were wiped gently. The GES-1 cells below the upper chamber were fixed with methanol and stained with crystal violet. The number of cells passing through the polycarbonate membrane was observed under a fluorescence microscope.

### Effect of F.AOH on apoptosis of ethanol-induced epithelial cells

GES-1 cells were seeded into six-well plates with 1 × 10^5^ cells in each well. After 24 h, the ethanol group was cultured with 7% ethanol for 4 h to induce cell damage. The drug groups were then cultured with F.AOH (2, 4 and 8 μg/mL) for 16 h, and all the group cells were collected. Annexin V-FITC/PI staining solution (Shanghai Yisheng Biotechnology, Shanghai, China) was added as instructed, and apoptosis was detected using Agilent flow cytometry (NovoCyte Quanteon, San Diego, CA).

### RNA extraction and RNA-seq

The normal group, acetic acid model group and F.AOH middle dose group were distributed with three rats in each group. The total RNA was extracted using Eastep^®^ Super Total RNA Extraction Kit (YEASEN, Shanghai, China). The concentration and purity of RNA were detected by NanoDrop 2000 ultramicro spectrophotometer (Thermo Fisher Scientific, Wilmington, DE). The agarose gel electrophoresis test detected RNA integrity, and RNA integrity (RIN) was determined by Agilent 2100 biological analyzer (Agilent Technologies, Santa Clara, CA). After mRNA was isolated from the total RNA and enriched by adding fragmentation buffer, the small fragments of the total mRNA were randomly broken and separated. The first-strand cDNA was synthesized by reverse transcriptase and random primers (K1621, Thermo Fisher Scientific, Wilmington, DE), followed by two-strand synthesis. After forming a stable double-stranded structure, End Repair Mix was added, and then an ‘A’ base was added at the 3′ end to connect the Y-shaped joint to nearly repair the end and connect the joint. The cDNA library was enriched by PCR and quantified by TBS380. The clusters were amplified by bridge PCR through cBot library and sequenced using Illumina Novaseq 6000 platform (Shanghai Meiji Biology, Shanghai, China) to generate the original data.

### Establishment of cDNA library and screening of differential genes

High-quality sequencing data (clean data) were filtered and mapped to *Rattus_norveicus* (RNOR_6.0) (http://www.ensembl.org/Rattus_norvegicus/Info/Index). The sample gene/transcript readings per million segments per kilogram base were analysed using quantitative software (RSEM). Then, millions of readings (TPM) obtained the standardized gene expression. Then, differentially expressed genes among gene sets by DESeq2 analysis (defining screening conditions *p* value <0.05 and fold change of differentially expressed genes (FC) ≥2 or FC <0.50) were screened.

### Gene function analysis

To better reflect the biological signals and potential mechanisms of the gene sets between the two groups, we analysed GO (http://www.geneontology.org/) and KEGG (http://www.genome.jp/kegg/) pathways. The differentially expressed genes in the signal pathway were analysed using the STRING database’s protein–protein interaction (PPI) network (https://string-db.org/).

### Analysis of Western blot

After the gastric tissue and cells were lysed by protein lysate, the proteins were extracted, and their concentrations were determined by the bicinchoninic acid protein analysis kit (Beyotime, Nanjing, China). Proteins were separated by sodium dodecyl sulphate-polyacrylamide gel electrophoresis and transferred to polyvinylidene fluoride membranes (MilliporeSigma, Burlington, MA). Membranes with 5% bovine serum albumin solution were sealed at room temperature for 1 h and then with the corresponding primary antibody (Table 1) at 4 °C for one night. On the second day, they were washed six times with TBST (tris-buffered saline and Tween 20) for 5 min, then incubated with horseradish peroxidase-labelled secondary antibody for 1.5 h, and washed again with TBST six times for 5 min each time. A Gel imaging system (Bio-Rad, Hercules, CA) and chemiluminescence detection reagent (Beyotime, Nanjing, China) were used to detect specific bands. Image analysis software (National Institutes of Health, Bethesda, MD) was used for quantitative analysis.

**Table 1.** The corresponding antibody name, manufacturer and dilution multiple list in Western blot experiment.

Antibody name	Manufacturer	Dilution multiple
Anti-endothelin 1 antibody	Abcam (ab2786)	1:1000
Anti-iNOS antibody	Beyotime(AF7281)	1:1000
Anti-Bax antibody	Beyotime (AF1270)	1:2000
Anti-Bcl-2 antibody	Abcam (ab32124)	1:1000
Anti-caspase-3 antibody	Beyotime (AF1213)	1:2000
Anti-EGF antibody	Abcam (ab218831)	1:2500
Anti-TRPV1 antibody	SANTA (sc-398417)	1:500
Anti-TLR4 antibody	Abcam (ab217274)	1:1000
Anti-NF-κBp65 antibody	Abcam (ab16502)	1:1000
Anti-phospho-IKB alpha	CST (2859T)	1:1000
Anti-beta actin antibody	Abcam (ab8227)	1:1000
Goat anti-rabbit IgG H&L (HRP)	Abcam (ab97051)	1:20,000

### Analysis of quantitative real-time PCR

Total RNA was extracted from gastric tissue and cells using Eastep^®^ Super Total RNA kit and total RNA was reverse transcribed using Hifair^®^II 1st Strand cDNA Synthesis SuperMix (YEASEN, Shanghai, China). Agilent’s Mx 3005p qPCR system amplification program (Agilent Technologies, Santa Clara, CA) was performed at 95 °C pre-denatured 5 min (one cycle) and 95 °C denaturation 10 °C annealing/extension 30 s (40 cycles). The results were analysed using the cyclic threshold (CT) method. All primer sequences are listed in Table 2.

**Table 2.** The mRNA primer sequence list for real-time quantitative PCR (qRT-PCR) in the experiment.

Primers	Forward/reverse	Sequence
Bcl-2	Forward	5′-CTGGTGGACAACATCGCTCT-3′
	Reverse	5′-ATAGTTCCACAAAGGCATCCCAG-3′
Bax	Forward	5′-GGTTTCATCCAGGATCGAGCA-3′
	Reverse	5′-TCCACATCAGCAATCATCCTCTG-3′
Caspase-3	Forward	5′-TTGGAACGGTACGCGAAGAA-3′
	Reverse	5′-AGAGTCCATCGACTTGCTTCC-3′
OSM	Forward	5′-TTAGTTTGGCCCTTGCGCT-3′
	Reverse	5′-GTTTTGGTGGAGGATATAGGGCT-3′
PDGFRB	Forward	5′-ACTATGTCCCATCTGCCCCT-3′
	Reverse	5′-ACTTGCCCTCACAGATGAGC-3′
CSF3R	Forward	5′-AGCCCCAATCATTGCTGAGA-3′
	Reverse	5′-AGCTTGGTGTTTACCTGCCT-3′
EGF	Forward	5′-TGCCAACTGGGGGTGCACAG-3′
	Reverse	5′-CTGCCCGTGGCCAGCGTGGC-3′
FGF18	Forward	5′-GCATCCATGTGGAGAACCAGA-3′
	Reverse	5′-CCACTAGGAGCTGGGCATACT-3′
SYK	Forward	5′-CCTGATGTGGGAAGCGTTCT-3′
	Reverse	5′-GGCCTGTTCTCCACATCGTA-3′
PGF	Forward	5′-TGAGGAACCCCACCTGTGAT-3′
	Reverse	5′-CTTGTCCTTCCATGCCCCTT-3′
NGF	Forward	5′-GCATCGCTCTCCTTCACAGAG-3′
	Reverse	5′-ACATTACGCTATGCACCTCAGAGT-3′
SRC	Forward	5′-TGAGGCATGAGAAGCTGGTG-3′
	Reverse	5′-TGCCTGAAGCAATCTGAGCA-3′
P2RY2	Forward	5′-CTGTCGACTCAGCGCCAAA-3′
	Reverse	5′-CTGGAGGGCTCCGAGGT-3′
TRPV1	Forward	5′-AGGGAGATCCATGAACCCGA-3′
	Reverse	5′-AGCATGTCATGACGGTTAGGG-3′
HTR2A	Forward	5′-GGTCATCATGGCAGTGTCCC-3′
	Reverse	5′-AAGGCCACCGGTACCCATAC-3′
BDKRB2	Forward	5′-ATACCGTGACCAGGACTTGAC-3′
	Reverse	5′-GTTGAACATTTCAATGCAGGTG-3′
CALCA	Forward	5′-CCTTTGAGGTCAACCTTGG-3′
	Reverse	5′-ATAGTTCTGCACCAGTGCA-3′
LBP	Forward	5′-ATGGAGATTGAAGGCTTTGTG-3′
	Reverse	5′-GAATACATTCGTGACCACGC-3′
CD14	Forward	5′-CGACCATGAAGCTTATGCTC-3′
	Reverse	5′-GAGAAGTTGCAGTAGCAGC-3′

### Statistical analysis

All experimental data were expressed by mean ± standard deviation, and statistical significance was analysed using GraphPad Prism 8.0 software (La Jolla, CA) with one-way analysis of variance (ANOVA). Tukey carried out the significant difference test. *p* Values <0.05 indicated statistical significance.

## Results

### Identification of the composition and structure of F.AOH

**Figure 1.**
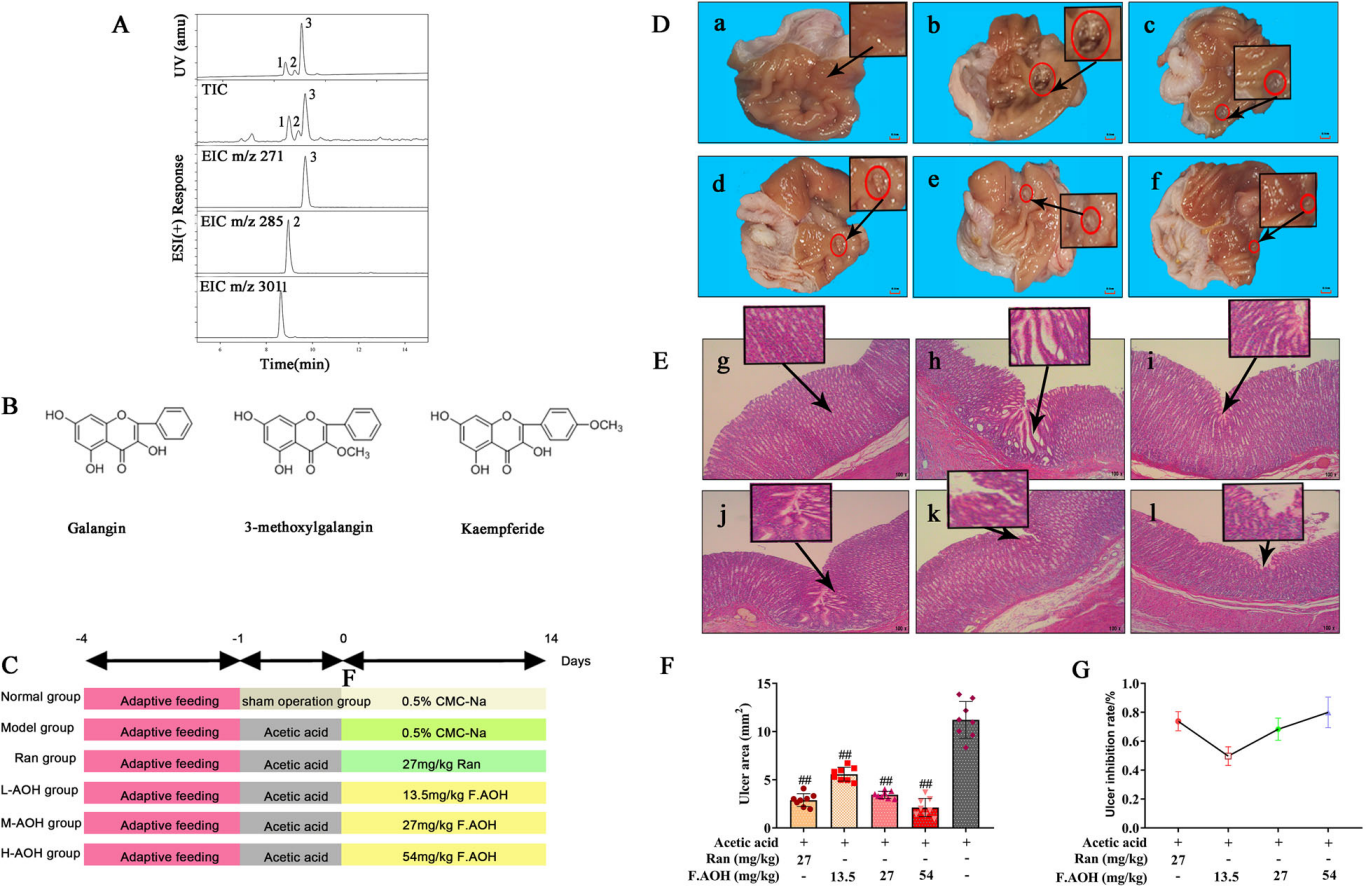
Composition and structure of F.AOH. (A, B) (Lin et al. 2020) macroscopic appearance and histological evaluation of gastric mucosa in rats with acetic acid-induced gastric mucosal damage (D, E), (a, g) normal group; (c, i) positive control group ranitidine (100 mg/kg); (d, j) F.AOH low- (13.5 mg/kg); (e, k) medium- (27 mg/kg); (f, l) high-dose (54 mg/kg); (b, h) model group treated with acetic acid; flowchart of experiments (C); F.AOH can increase the ulcer inhibition rate and decrease ulcer index in rats with GU (F, G). ^##^*p* < 0.01 compared with model group (*n* = 6).

By analysing ultra-high performance liquid chromatography diode array detector and mass spectrometer, we found three kinds of compound monomers in F.AOH: (a) galangin: 3,5,7-trihydroxy-flavone; (b) 3-methyl galangin: 5,7-dihydroxy-3-methoxy-flavone; (c) kaempferide: 3,5,7-trihydroxy-4-methoxy-flavone (Figure 1(A,B)) (Lin et al. 2020).

### F.AOH by oral administration prevented by acetic acid-induced chronic gastric ulcer

We scored the UI of gastric tissue according to Equation (1), as displayed in Figure 1(F,G). Round ulcers appeared in all rats stimulated by acetic acid, while the sham operation group showed a normal state. The ulcer area of rats cured with F.AOH or RAN was less than that of the acetic acid model group, and the effect of the high dose group was better than that of the RAN group (Figure 1(D)). Next, we used HE staining to observe the pathology of the ulcer area, as depicted in Figure 1(E). Compared to the normal group, the acetic acid model group showed inflammation, neutrophil aggregation, more vesicles in the ulcer area, tissue proliferation and obvious cystic hyperplasia, showing part of intestinal metaplasia consistent with the pathology of clinical chronic GU. After drug treatment, the pathological changes in gastric tissue were improved.

### F.AOH inhibits the level of ET-1-related inflammatory factors by increasing NO content

**Figure 2.**
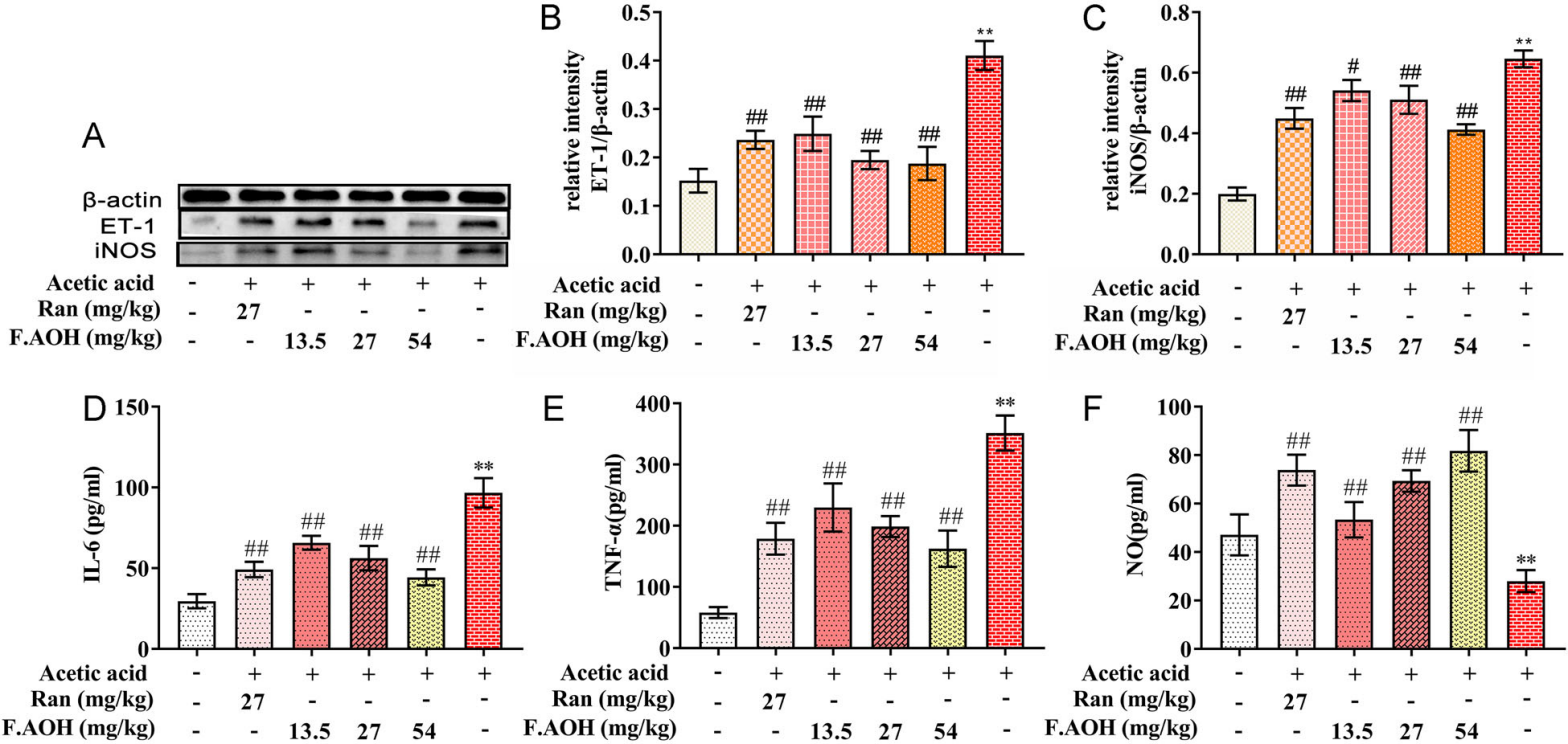
The levels and expressions of endothelin-related inflammatory factors (A–C), the expression levels of nitric oxide synthase, and ET-1 proteins were analysed; (D–F) the levels of inflammatory factors NO, IL-6 and TNF-α in serum. **p < 0.01 compared with normal group; ^#^*p* < 0.05 and ^##^*p* < 0.01 compared with model group (*n* = 6).

In analysing the association between chronic GUs and endotoxin-associated inflammation induced by acetic acid, ELISA analysis revealed that serum levels of inflammatory factors IL-6 (Figure 2(D)), TNF-α (Figure 2(E)) and NO (Figure 2(F)) were significantly higher in the acetic acid-stimulated group compared to the normal group. In addition, the expression of ET-1 and iNOS protein in gastric tissue was significantly high. On the contrary, with the increase in dose, F.AOH inhibited the level of serum inflammatory factors more significantly and decreased the expression of ET-1 and iNOS in a dose-dependent manner (Figure 2(A–C)).

### Oral administration of F.AOH ameliorated the severity of oxidative stress in GU rat model

The dynamic imbalance of ET-1 and NO can aggravate oxidative stress (Tarnawski et al. 2012). Next, we evaluated the effects of F.AOH on oxidative stress and gastric protection. We used biochemical indexes such as MPO, SOD, GSH and MDA to detect the process of oxidative stress. As presented in Table 3, compared to the normal group, acetic acid induction can cause significant changes in the biochemical indexes of oxidative stress, resulting in lipid peroxidation and tissue damage aggravation. The serum SOD activity and GSH level in F.AOH and RAN group were significantly higher than those in the acetic acid model group but lower than those in the normal group. In contrast, MPO activity and MDA level were significantly lower than those in the acetic acid model group but higher than those in the normal group.

**Table 3.** F.AOH attenuates oxidative stress in rats with gastric injury induced by acetic acid.

Group	MPO (U/mL)	SOD (U/mL)	GSH (nmol/mL)	MDA (nmol/mL)
Normal	1.52 ± 0.67	17.62 ± 0.98	41.6 ± 4.8	15.74 ± 1.37
RAN (100 mg/kg)	13.45 ± 1.02#	10.4 ± 0.7##	26.43 ± 6.45#	49.61 ± 3.63##
F.AOH (31.7 mg/kg)	11.28 ± 1.06#	6.9 ± 1.26	28.1 ± 5.2#	61.42 ± 6.2#
F.AOH (63.4 mg/kg)	8.47 ± 3.47##	8.31 ± 1.7#	30.3 ± 3.4##	41.7 ± 7.83##
F.AOH (126.8 mg/kg)	6.15 ± 0.84##	13.28 ± 1.54##	33.41 ± 4.38##	33.76 ± 3.84##
Acetic acid model	17.72 ± 1.42**	5.6 ± 1.2**	18.8 ± 3.46**	98.65 ± 5.52**

**p < 0.01 compared with normal group; ^#^*p* < 0.05 and ^##^*p* < 0.01 compared with model group (*n* = 6).

### F.AOH promoted the proliferation, migration and inhibition of apoptosis of GES-1 cells

**Figure 3.**
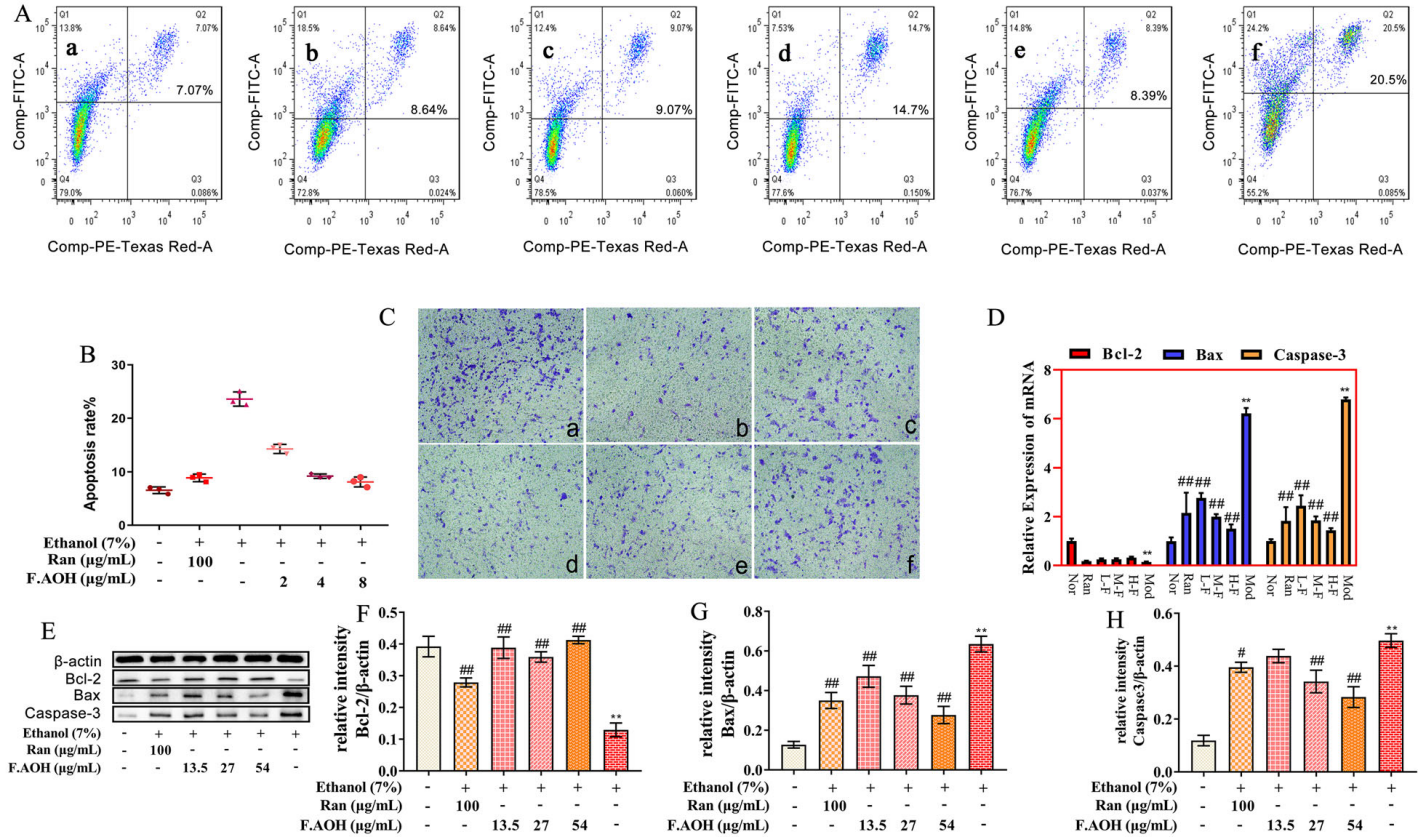
F.AOH promoted proliferation, migration and inhibiting apoptosis of GES-1 cells *in vitro*. (A, B) Flow cytometry and apoptosis rates for each group: (a) normal group, (b) positive control group ranitidine, (c) F.AOH low- (13.5 mg/kg), (d) medium- (27 mg/kg), (e) high-dose- (54 mg/kg), (f) model group treated with ethanol; (C) F.AOH can promote the penetration rate of GES-1 cells in transwell test: (a) normal group, (b) model group treated with ethanol, (c) positive control group ranitidine, (d) F.AOH low- (13.5 mg/kg), (e) medium- (27 mg/kg), (f) high-dose- (54 mg/kg); (D–H) F.AOH inhibiting the expression of Bcl-2/Bax/caspase-3 pathway proteins and mRNA in GES-1 apoptosis *in vitro*. *p < 0.05 and **p < 0.01 compared with normal group; ^#^*p* < 0.05 and ^##^*p* < 0.01 compared with model group.

The protective effects of GU include gastric epithelial renewal, including but not limited to apoptosis, repair and proliferation of GES-1 cells. Therefore, we evaluated the effects of ethanol on gastric epithelial cell injury, proliferation, apoptosis, and the protective effect of F.AOH treatment. Flow cytometry revealed that the ethanol-induced apoptosis rate of GES-1 cells was significantly higher than that of the normal group (Figure 3). Compared with the ethanol model group, F.AOH treatment significantly reduced the apoptosis rate of GES-1 cells (Figure 3(A,B)). Western blotting and qPCR experiments disclosed that F.AOH augmented Bcl-2 protein and gene expression while decreasing Bax and caspase-3 protein and gene expression (Figure 3(D–H)). In addition, the results displayed that F.AOH treatment significantly resisted the damage degree of gastric mucosal epithelial cells in ethanol and increased the number of cells passing through the polycarbonate membrane (Figure 3(C)).

### Effect of F.AOH on the mRNA expression profile

**Figure 4.**
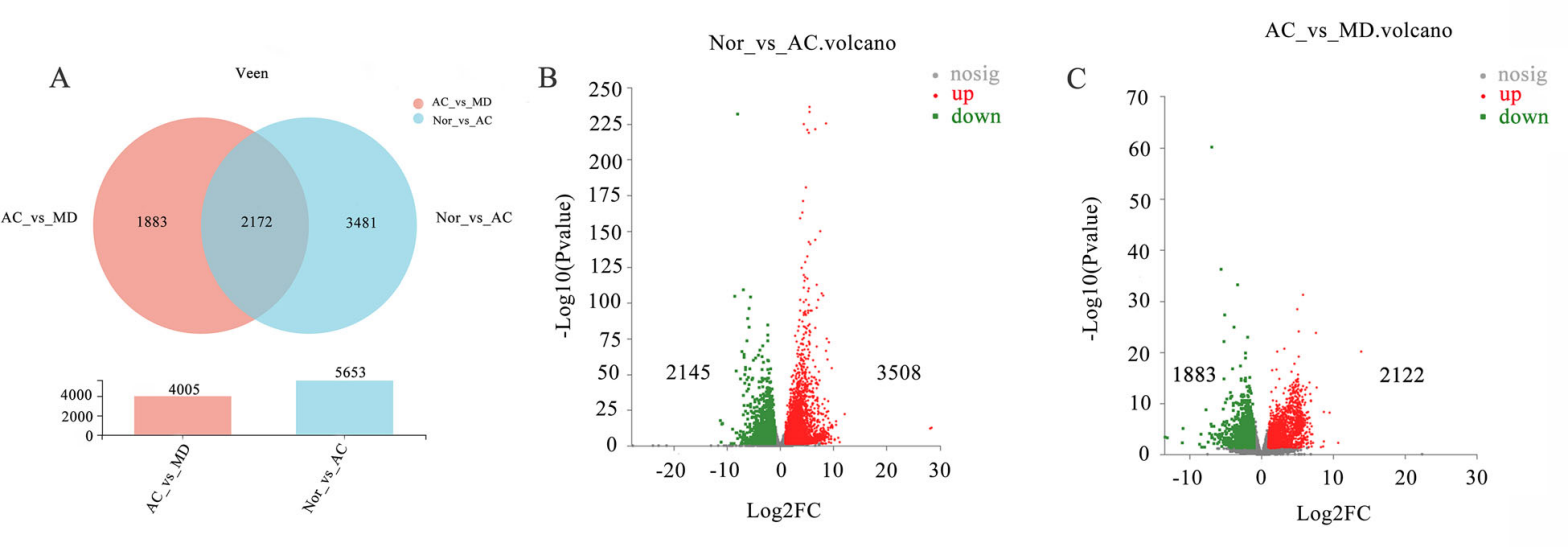
(A) Venn maps of differential gene expression. The volcanic map of (B) NOR vs. AC and (C) AC vs. MD (red dots are upregulated genes, blue dots are upregulated genes and grey dots are non-significant differential genes).

To further understand the potential pathogenesis of GU and the role of F.AOH in treating GU, RNA-seq was performed in GU region. As manifested in Figure 4, the total gene (32883 mRNA) was differentially altered in 17.2% (5653 mRNA) of acetic acid-induced gastric tissue. Compared with the normal group, 3508 gene differences were upregulated, and 2145 were down-regulated in the acetic acid group. Concurrently, about 12.2% (4005 mRNA) of genes were significantly changed in F.AOH group compared with the acetic acid group, with 2122 genes differentially up-regulated and 1883 genes differentially up-regulated. In addition, the gene sets of the three groups were highly concentrated and expressed in similar regions.

### GO enrichment analysis

**Figure 5.**
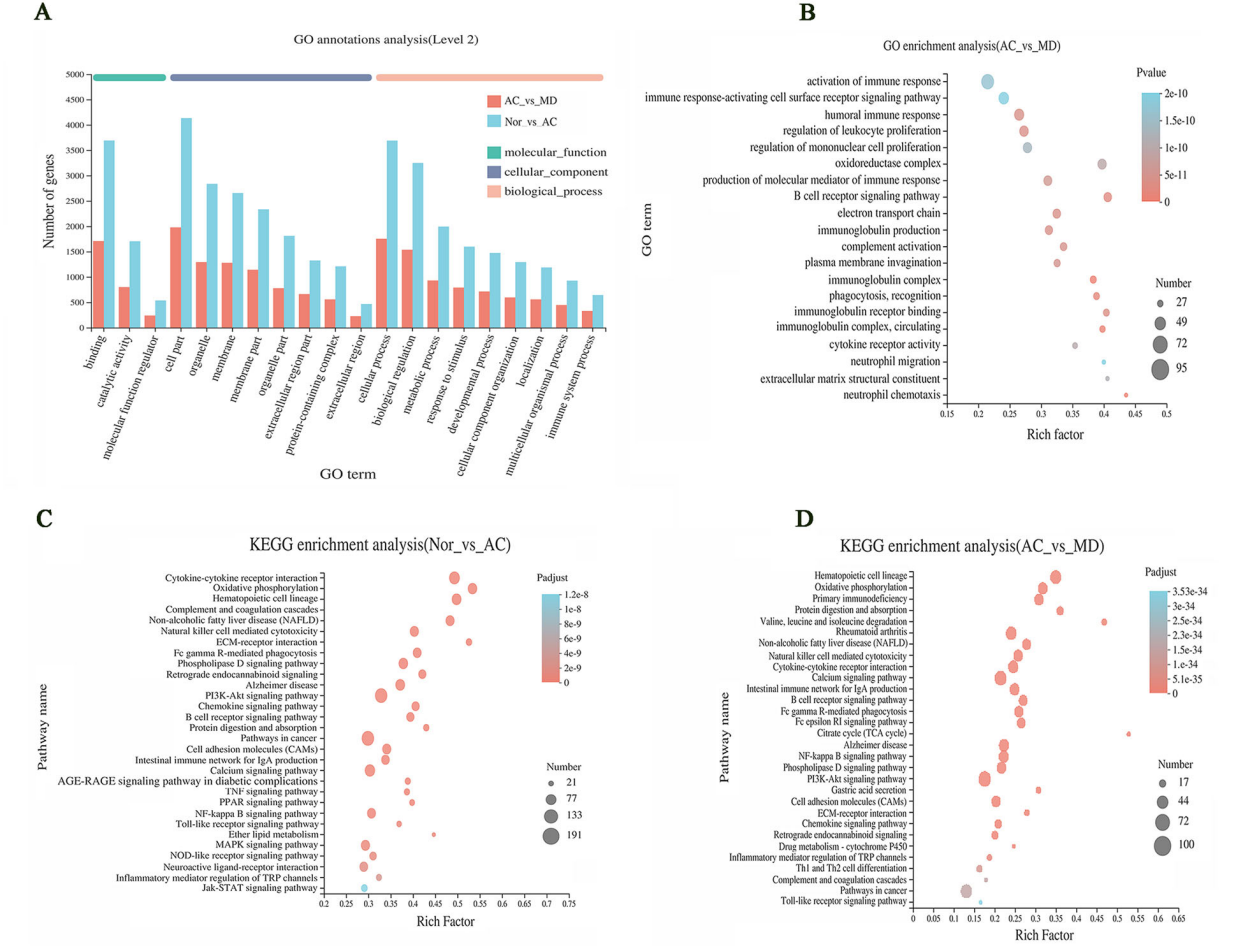
The map of GO enrichment and KEGG pathway enrichment analysis of differential genes. (A, B) GO functional enrichment analysis map of NOR vs. AC and AC vs. MD (single gene bubble map of the top 20 enrichment abundance); (C, D) the KEGG functional enrichment analysis map of NOR vs. AC and AC vs. MD (single gene set bubble map of the top 30 enrichment abundance).

The DEGs obtained from the NOR vs. AC and AC vs. MD genomes were enriched in GO function from biological processes, molecular functions and cell composition. These aspects were used to analyse the related functional pathways that DEGs might be involved in regulation. They mainly involve cell part, binding, cellular process, biological regulation, response to stimulus, and metabolic processes (Figure 5(A)). As revealed in Figure 5(B), GO enrichment analysis is at the top 20 GO enrichment of the AC vs. MD gene set. It mainly involves activation of immune response, oxidoreductase complex, regulation of leukocyte proliferation, immunoglobulin complex, production of molecular mediator of immune response and other pathways. This suggests that F.AOH may improve or inhibit the process of GUs by regulating the function of the immune response, participating in the oxidative stress, protecting the integrity of gastric mucosa, and regulating cell adhesion.

### KEGG pathway enrichment analysis

The enrichment analysis of KEGG pathway found that DEGs of NOR vs. AC and AC vs. MD gene could be significantly enriched to 329 and 316 signal pathways. The single genome (bubble plot) condenses the first 30 signalling pathways shown (Figure 5(C,D)). The results displayed that GU was mainly involved in signalling pathways including but not limited to PI3K-Akt and cytokine–cytokine receptor interaction (Figure 5(C)). In addition, the significantly different genes in AC vs. MD group were mainly enriched in the calcium, PI3K-Akt, NF-κB and other signalling pathways (Figure 5(D)). Notably, many common signalling pathways were regulated in GU and drug treatment.

### Three KEGG signal pathways of DEGs affected by F.AOH

**Figure 6.**
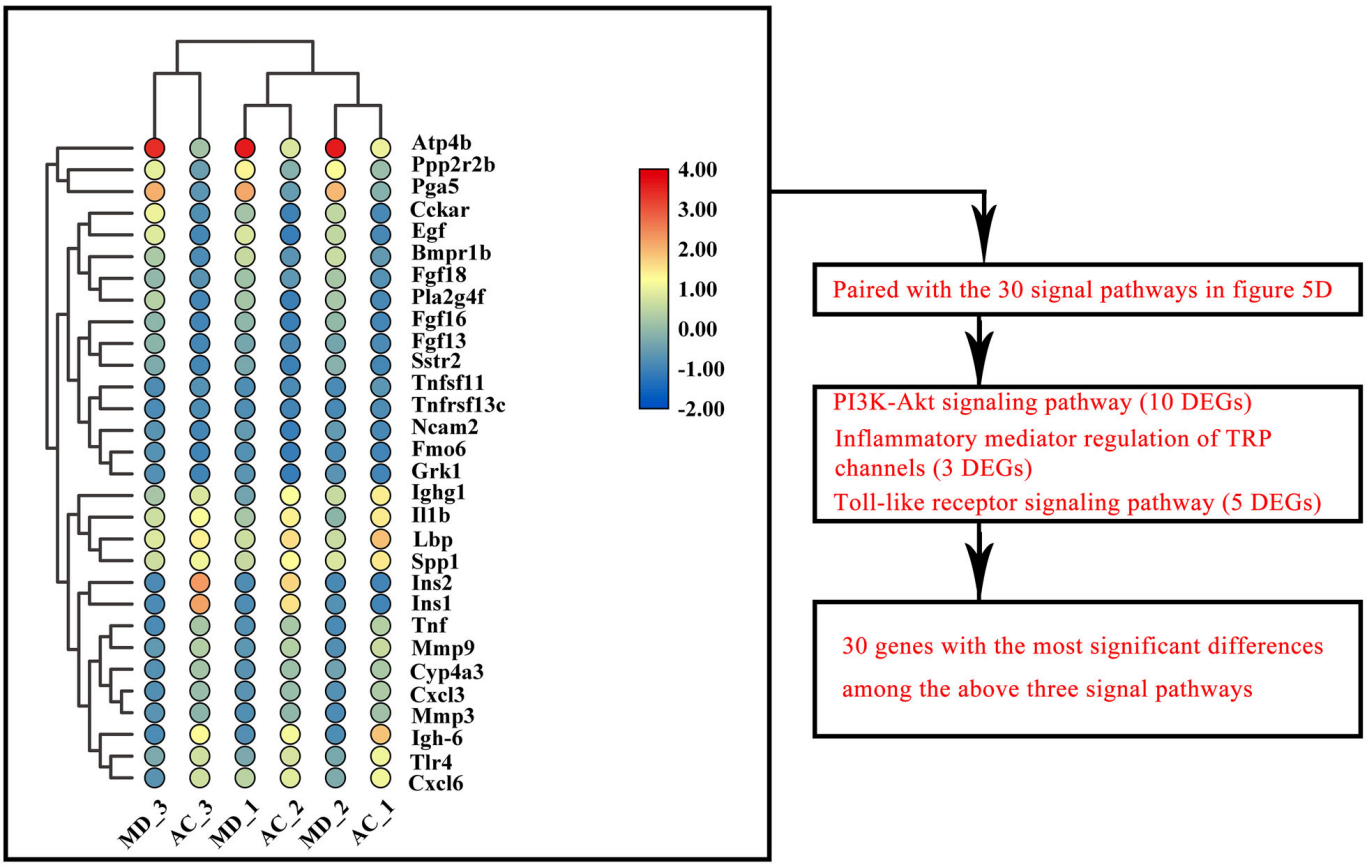
The 30 genes with the most significant differences among all differential genes in the first 30 signalling pathways enriched by KEGG of AC vs. MD.

All differential genes of the top 30 signalling pathways enriched by AC vs. MD were screened for the 30 genes with the most significant differences (Figure 6). Three enrichment signal pathways of interest were screened by pairing these 30 genes with the above 30 signal pathways. These are PI3K-Akt signal pathway (including 10 DEGs), inflammatory mediator regulation of TRP channels (including three DEGs) and Toll-like receptor signal pathway (including five DEGs) to verify that F.AOH can play a role in GU treatment and protection by regulating multiple signal pathways (Figure 5(D)).

### Identification of key genes by PPI network and verification of Western blot and qPCR

**Figure 7.**
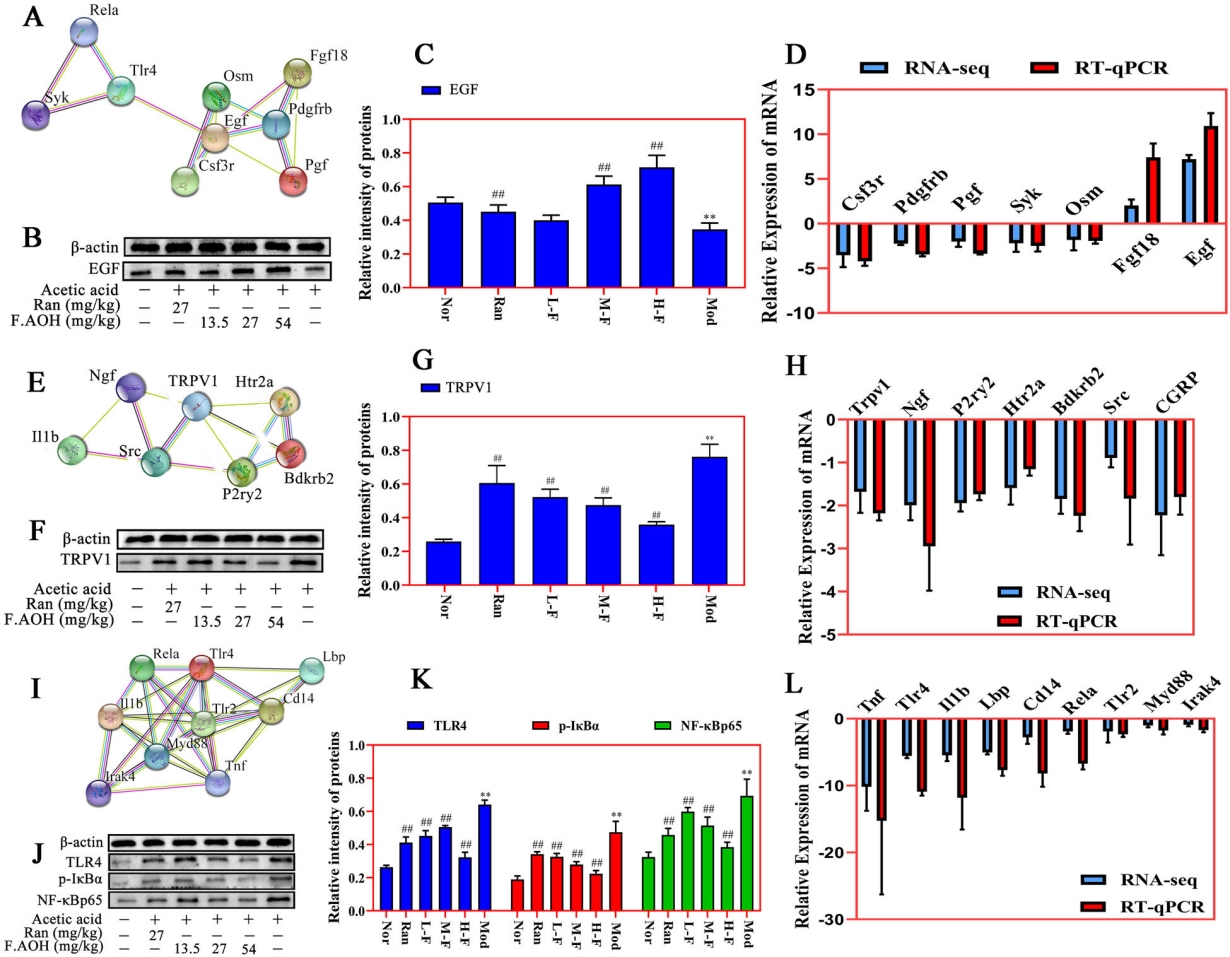
PPI network recognition of key genes and Western blot and qPCR verification experiments. (A–L) PI3K-Akt pathway, Toll-like receptor pathway and inflammatory mediators regulate the relative expression levels of TRP channel-related genes and proteins. **p < 0.01 compared with normal group; ^##^*p* < 0.01 compared with model group: values are expressed as means ± standard deviation (*n* = 3).

We mapped the most significant heatmaps of differential genes in 30 of the three signalling pathways and selected several key genes into STRING database (Figure 7(A,E,I)). Western blot and qPCR were used to verify the relationship between gene expression and protein interaction (Figure 7). The results presented that acetic acid-induced GU was associated with Toll-like receptor activation, regulation of TRP channel by inflammatory mediators, and expression regulation of some key genes and proteins in the PI3K-Akt signalling pathway. F.AOH treatment can activate or inhibit the expression levels of related genes and proteins, promoting gastric protection. In addition, qPCR was performed on the key genes selected by the three signalling pathways, and gene expression trends were compared with those obtained by mRNA-seq. qPCR results were consistent with RNA-seq (Figure 7(D,H,L)).

## Discussion

Herein, we analysed the potential molecular mechanism of acetic acid-induced GU in rats, evaluated the anti-ulcer effect of F.AOH, and further explored its potential functional mechanism based on previous experimental results (Lin et al. 2020). Our experiments revealed that gastric mucosal protective dysfunction, immune response and sensory nervous system conduction are involved in regulating and developing GU. Although RAN, a positive control drug, has been widely used in the clinical treatment of GU, previous studies have reported its side effects, including renal adverse reactions and osteoporosis (Yu et al. 2017). Our results display that medium-dose (27 mg/kg) and high-dose (54 mg/kg) F.AOH shows similar effects or significant advantages over RAN in GU treatment (Figures 1–3, Table 1). Therefore, F.AOH may be a potential novel and effective supplementary drug for GU treatment. Importantly, transcriptome sequencing revealed that F.AOH could enhance the function of the immune system by mobilizing multiple molecular signalling pathways in the body, reducing the biological cascade reaction caused by the onset of GU, and ultimately accelerating the healing and pathologically improving GU.

Endothelin is considered one of the important ulcer factors, which can cause severe vasoconstriction of gastric mucosa, resulting in insufficient gastric blood supply, local hypoxia and aggravating oxidative stress (Tarnawski et al. 2012; Oncel and Basson 2022). Our experiments demonstrate that OH can regulate endothelin associated with inflammation, reduce endothelin secretion, increase nitric oxide synthase activity and promote the production of nitric oxide (Figure 2). Accordingly, we speculated that well within the scope of influence and dynamic balance, ulcer tissue endothelin and nitric oxide improve the activity of SOD and GSH, lower MPO enzyme activity, decrease MDA release (Table 1) and improve oxidative inflammatory response induced by stress.

High-throughput sequencing is a transcriptome sequencing technique. Through sample preparation, library establishment, mRNA sequencing operation and data screening and analysis, the mechanism of candidate genes or potential molecular signal pathways are identified (Hrdlickova et al. 2017; Yang et al. 2020). According to GO enrichment analysis (Figure 5(B)), we speculated that F.AOH might improve or inhibit GU occurrence by enriching and regulating immune response, participating in the oxidative stress process, protecting the integrity of gastric mucosa, and regulating cell adhesion. Furthermore, based on KEGG pathway enrichment analysis results (Figure 5(D)), we speculated that F.AOH could improve the immune function of rats, control metabolic disorders, enhance the information transmission of biological cascade reactions, regulate cell proliferation, migration, apoptosis, protect the integrity of gastric mucosa, and prevent GU from developing to severe or even canceration. Then, Toll-like receptors signalling pathways, regulation of inflammatory mediators TRP channels, and PI3K-Akt signalling pathway in several key genes and proteins for validation were chosen. Finally, we found FAOH treatment can promote GU anti-inflammatory analgesic action, promote healing of ulcers of tissue repair, and epithelial cell proliferation and migration to improve the cure rate of GU. The pathological mechanism of these imbalances is closely related to GU occurrence and development (Tarnawski and Ahluwalia 2012).

**Figure 8.**
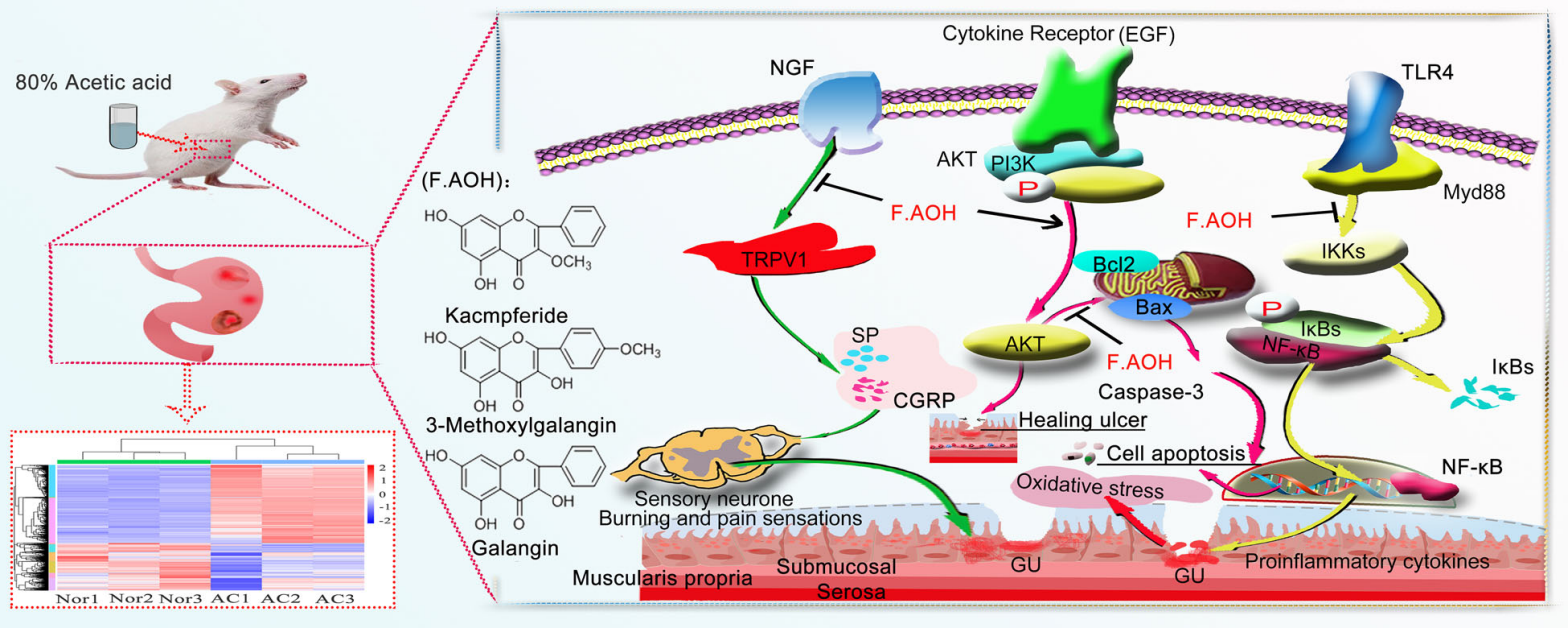
F.AOH is involved in TLR4/NF-κB and TRPV1 signalling pathways and gastric mucosal ulcer healing.

PI3K-Akt signalling pathway is induced by serine or threonine in downstream substrates through autophosphorylation and receives cell signals, mediating biological growth and metabolism, vascular remodelling, and wound healing (Ersahin et al. 2015). The regulation of certain genes and molecular proteins in this pathway leads to the increase or loss of cell development function, and GU is closely related to this pathway (Tarnawski and Ahluwalia 2021). GU healing and repair is the reconstruction of the muscle layer and structure of gastric mucosa. After extracellular matrix degradation, proliferation and migration of covering the ulcer area, the formation of granulation tissue, then under the stimulus growth factor and OSM (oncostatin M) deep ulcer to the epithelial mucous membrane and the defect of filling the connective tissue and rebuild microvascular blood oxygen nutrition and energy for ulcer healing, in addition to SYK (spleen tyrosine kinase) influence this process (Ruzza et al. 2009; Tarnawski and Ahluwalia 2012; Tarnawski and Ahluwalia 2021). The result may be an abnormal cascade reaction, in which the enlargement of inflammatory response leads to GU enlargement or necrosis of epithelial cells, affecting the ulcer healing process. Our transcriptome and experimental data show that some genes and proteins in PI3K-Akt pathway are involved in the repair of GU in rats when F.AOH is present (Figure 8(A–D)).

Toll-like receptor signalling pathways are widely distributed in immune cells and play an important role in recognizing specific pathogen molecular patterns and initiating inflammatory immune responses to pathogens (Fitzgerald and Kagan 2020). TLR2 and TLR4 are mostly under expressed or silenced in TLR family, but when inflammation occurs, they can pass through MYD88. The action of junction molecules such as IRAK4 TRAF6 promotes the activation of the transcription factor NF-κB and induces cytokine secretion and inflammatory responses (Hu et al. 2021; Pereira et al. 2022). This study found that acetic acid activated the expression of some genes and proteins in the Toll-like receptor signalling pathway. On the contrary, F.AOH treatment significantly inhibited the activation of some Toll-like receptor signalling pathways and regulated the expression of related genes and proteins. Based on this, we concluded that F.AOH treatment could block TLR4/MYD88/NF-κB signalling pathway, thereby affecting its adhesion chemotaxis, proliferation, activation and activation. Apoptosis and secretion of inflammatory cytokines promote gastric protection (Figure 7(E–H)).

The pain effect is one of the reasons that makes people suffer to bear the onset of GU. The studies have found that many diseases related to inflammatory pain are correlated with the increase of endogenous NGF (Barker et al. 2020; Wise et al. 2021). Transient receptor potential cationic channel subfamily V1 (TRPV1) can mediate nociceptive receptor activation and thermal pain sensitization. TRPV1 binds to SRC kinase and is phosphorylated by tyrosine, leading to sensitization of TRPV1, while the intervention with SRC kinase inhibitor PP2 reduces NGF phosphorylation of TRPV1 (Robilotto et al. 2022). P2Y G protein-coupled nucleotides are critical for stimulating nociceptive sensory neurons by continuously modulating TRPV1 function. P2Y deficient mice significantly reduced inflammatory heat pain conduction and TRPV1 sensitivity (Malin et al. 2008). Finally, sensitization and activation of TRPV1 release inflammatory-related neurotransmitters or neuropeptides, such as calcitonin gene-related peptide (CGRP, which has strong vasodilating effects) and substance P (SP, which is associated with pain transmission) (Quartu et al. 2016; Shuba 2020). The endogenous active substance 5-hydroxytryptamine receptor plays an important role in neurogenic inflammation and nociceptive sensitization (Su et al. 2016; Guzel and Mirowska-Guzel 2022). We verified the involvement of acetic acid in the activation of part of the cascade reactions of the above pathways by transcriptional sequencing, qPCR and Western blot experiments. Simultaneously, we found that F.AOH antagonizes the negative effect of acetic acid on rats, blocks the partial activation of inflammatory mediators regulating TRP channels, and shows the pharmacological activity of regulating the pain of an inflammatory ulcer, thus relieving neuralgia in rats with chronic GU (Figure 7(I–L)).

## Conclusions

Our study further demonstrates that F.AOH could be employed as an anti-GU plant component as it has involved in TLR4/NF-κB and TRPV1 signalling pathways and gastric mucosal ulcer healing. This provides candidate target pathways and strategies for the treatment of GU by F.AOH (Figure 8), and suggests that F.AOH has potential therapeutic value in the treatment of gastrointestinal diseases such as anti-inflammatory pain and antioxidant stress. However, given the current research results, the potential effective therapeutic approaches and targets of drugs deserve further verification and disclosure.
